# Proteome Analysis and Epitope Mapping in a Commercial Reduced-Gluten Wheat Product

**DOI:** 10.3389/fnut.2021.705822

**Published:** 2021-08-11

**Authors:** Mitchell G. Nye-Wood, Angéla Juhász, Utpal Bose, Michelle L. Colgrave

**Affiliations:** ^1^School of Science, Edith Cowan University, Perth, WA, Australia; ^2^Australian Research Council Centre of Excellence for Innovations in Peptide and Protein Science, Perth, WA, Australia; ^3^Commonwealth Scientific and Industrial Research Organisation (CSIRO) Agriculture and Food, St Lucia, QLD, Australia

**Keywords:** gluten, wheat, celiac disease, allergy, food safety, proteomics, mass spectrometry

## Abstract

Gluten related disorders, such as coeliac disease, wheat allergy and baker's asthma are triggered by proteins present in food products made from wheat and related cereal species. The only treatment of these medical illnesses is a strict gluten-free diet; however, gluten-free products that are currently available in the market can have lower nutritional quality and are more expensive than traditional gluten containing cereal products. These constraints have led to the development of gluten-free or gluten-reduced ingredients. In this vein, a non-GMO wheat flour that purports to contain “65% less allergenic gluten” was recently brought to market. The present study aims to understand the alteration of the proteome profile of this wheat flour material. Liquid chromatography-mass spectrometry was used to investigate the proteome profile of the novel wheat flour, which was contrasted to a wheat flour control. Using both trypsin and chymotrypsin digests and a combined database search, 564 unique proteins were identified with 99% confidence. These proteins and the specific peptides used to identify them were mapped to the wheat genome to reveal the associated chromosomal regions in the novel wheat flour and the mixed wheat control. Of note, several ω- and γ-gliadins, and low-molecular weight glutenins mapping to the short arm of chromosome 1, as well as α-gliadins from the chromosome 6 short arm were absent or expressed at lower levels in the novel wheat variety. In contrast, the high-molecular weight glutenins and α-amylase/trypsin inhibitors were notably more abundant in this variety. A targeted quantitation experiment was developed using multiple reaction monitoring assays to quantify 359 tryptic and chymotryptic peptides from gluten and related allergenic proteins revealing a 33% decrease of gluten protein content in the novel wheat flour sample in comparison to mixed wheat control. However, additional mapping of known allergenic epitopes showed the presence of 53% higher allergenic peptides. Overall, the current study highlights the importance of proteomic analyses especially when complemented by sequence analysis and epitope mapping for monitoring immunostimulatory proteins.

## Introduction

Wheat products account for some 20% of dietary calories and protein ingested globally (1). However, in susceptible people wheat proteins can elicit a range of health disorders including coeliac disease (CD), wheat allergy (WA), and non-coeliac wheat sensitivity (NCWS). The allergenic wheat proteins that cause these adverse immune reactions have been mapped to specific genes by The International Wheat Genome Sequencing Consortium (IWGSC) and collaborators (2, 3). CD is caused when dietary gluten reaches the small intestine of genetically predisposed individuals and stimulates an autoimmune response leading to localized damage and subsequent symptoms (4). WA, as well as baker's asthma (BA) and wheat-dependent exercise induced anaphylaxis (WDEIA), involve an IgE-mediated immune response to wheat proteins that are either ingested as food or occur via skin contact or inhalation. While these disorders can be triggered by gluten proteins, BA typically has a non-gluten protein trigger (5, 6). NCWS is diagnosed when symptoms develop in response to cereal grain consumption, but serological testing is negative for both an autoimmune response and the IgE-mediated allergic response, which contraindicates CD and WA, respectively (4, 7). While the term “non-celiac gluten sensitivity” has a history of use (8), NCWS better reflects the non-gluten wheat proteins (9) or non-protein wheat components like FODMAPs (10) that elicit similar symptoms (7). CD is estimated to afflict ~0.7–1.4% of the global population (11, 12), WA some 0.33–0.75% in adults (13–15), and NCWS being more variable but with prevalence estimated between 0.5 and 13% (16, 17). While gluten proteins are established antigens to those with CD and also contribute to various allergies, non-gluten wheat proteins are potential allergens and antigens capable of causing WA, BA, NCWS, as well as CD (3, 6, 18).

The only effective treatment for these wheat-related immune disorders is the exclusion of wheat and related crop species from the diet. This adds to demand for “gluten free” foodstuffs that resemble traditional wheat products, however wheat is replaced by substitute ingredients that contribute starch but without gluten or other cereal proteins. The absence of gluten proteins, however, can affect the consistency, texture, or taste of gluten-free products, and the substitute ingredients typically come at a higher cost and require recipe alterations (19).

Several approaches have aimed at reducing the gluten content while retaining the health benefits of whole grains or maintaining the unique functionality of cereal grains. One approach is to use ethyl methanesulfonate (EMS) mutagenesis to produce random mutations in genetic material by nucleotide substitution. EMS is often used as the technology base for “Targeting Induced Local Lesions in Genomes” (TILLING) which has proven effective at targeting key wheat enzymes to improve starch composition (20). It has been applied to wheat gluten genes (21), but is challenged by the sheer number of potential allergens and the fact that even low levels of expressed gliadins can elicit CD (22). In barley, ultra-low gluten levels (<5 mg/kg) were achieved in the variety Kebari^®^ by using traditional breeding techniques to combine mutagenesis-derived barley varieties with decreased hordein content and composition (23, 24). Efforts to develop a low-allergen wheat variety have targeted genes that either exhibit a large immune response directly (25), or that conduct epigenetic regulation of downstream gluten protein genes (26), and have also made use of natural null-allele variants (27), CRISPR-Cas9 (28), and RNAi (29). A common phenotype is that downregulation of one or a subset of gluten protein encoding genes is accompanied by the compensatory upregulation of alternate storage proteins (30–34), with a change in technological properties (35). However, technology to characterize gluten in wheat products is the subject of ongoing research (36), as is the targeted removal of CD reactive epitopes from wheat (37).

Recently, a reduced gluten product was released that claims to be a non-GMO wheat variety which contains “*65% less allergenic gluten than traditional flour*.” It is clarified that the product is “*developed for those with sensitive stomachs who don't have gluten or wheat allergies, but who want to reduce the amount of gluten in their diets*” (38) and was developed using wheat prolamin box binding factor (PBF) mutants (21, US patents 9,150,839, 10,412,909, and 10,750,690). There are no reports of the proteome and overall characteristics of proteins present in the reduced gluten flour in comparison to commercial wheat varieties.

Wheat has more than 800 genes with potentially allergenic domains, and some 356 genes encoding reference food allergens are included in the “IWGSC v1.0 reference allergen map” (3). This includes gliadins (including α-, β-, γ-, and ω-subtypes) and glutenins (including low molecular weight (LMW) glutenins and high molecular weight (HMW) glutenin subunits), as well as avenin-like proteins (ALPs), α-amylase/trypsin inhibitors (ATIs), and lipid transfer proteins (LTPs) (3). The gluten proteins contain specific epitopes that are deamidated, recognized, and presented by MHC-II antigen presenting cells in the gastrointestinal tract, in doing so initiating the autoimmune response that characterizes CD (39). The canonical gluten proteins, the gliadins and glutenins, together make up some 80% of the protein content in the wheat endosperm, and the most potent contributors to CD toxicity are the chromosome 6D α-gliadins and chromosome 1D ω-gliadins (ω 1,2 sub-type), followed by the LMW glutenins and γ-gliadin (40). It is therefore important to precisely characterize protein groups and epitopes when quantifying “allergenic gluten” in new products. This present study aimed to understand the alterations to the proteome in this reduced gluten wheat product using LC-MS/MS in comparison to a mixed wheat control.

## Materials and Methods

### Sample Preparation

GoodWheat^™^ (GW) white bread wheat flour was purchased directly from Arcadia Bioscience (Davis, CA, USA). Replicates of GW and of a mixed-wheat (MW) control flour sample were weighed out in quadruplicate. The MW control consisted of equal parts of flour from wheat cultivars: Alsen, Xiayan, Pastor, Westonia, Baxter, Chara, Yitpi, AC Barrie, and Volcania; selected to represent the diversity of wheat used in commercial production. Gluten proteins were specifically enriched from the wheat using an isopropanol/dithiothreitol (IPA/DTT) solvent as described previously (41). Flour (50mg) was weighed into a 1.5 mL micro-tube and 500 μL (10 μL/mg) of 55% IPA/2% DTT was added with vortex mixing until the flour was thoroughly combined with the solvent. The tubes were then sonicated for 5 min at room temperature and incubated in a thermo-mixer (400 rpm, 30 min, 50°C). The tubes were centrifuged for 15 min at 20,800 ×g. The solutions were centrifuged for 15 min at 20,800 ×g. Protein extracts (100 μL) were added to 10 kDa molecular weight cut off filters (Merck, Bayswater, Australia). The protein on the filter was washed twice with a buffer consisting of 8M urea in 0.1M Tris-HCl (pH 8.5) with centrifugation for 15 min at 20,800 ×g. Iodoacetamide (25mM; 100μL) prepared in 8 M urea and 100 mM Tris-HCl was added to the filters for cysteine alkylation with incubation in the dark for 20 min prior to centrifugation for 10 min at 20,800 ×g. The buffer was exchanged with 100 mM ammonium bicarbonate (pH 8.0) by two consecutive wash/centrifugation steps. The filters were transferred to fresh collection tubes and digestion enzyme, either trypsin or chymotrypsin (Promega, NSW, Australia), was prepared as 10 μg/mL in 100 mM ammonium bicarbonate, 50 mM calcium chloride and 200 μL was added to each filter with incubation for 16 h at 37°C. The filters were centrifuged for 15 min at 20,800 ×g. The filters were washed with 200 μL of 100 mM ammonium bicarbonate, and the combined filtrates were subsequently lyophilized.

### Discovery Proteomics

The digested samples were reconstituted in 100 μL of 1% formic acid and the peptides (1 μL) were chromatographically separated on an Ekspert nanoLC415 (Eksigent, Dublin, CA, USA) coupled to a TripleTOF 6600MS (SCIEX, Redwood City, CA, USA). The peptides were desalted for 5 min on a ChromXP C18 (3 μm, 120 Å, 10 × 0.3 mm) trap column at a flow rate of 10 μL/min of 0.1% formic acid and separated on a ChromXP C18 (3 μm, 120 Å, 150 × 0.3 mm) column at a flow rate of 5 μL/min. The solvents used were (A) 5% DMSO, 0.1% formic acid, 94.9% water and (B) 5% DMSO, 0.1% formic acid, 90% acetonitrile, 4.9% water. A linear gradient from 3 to 25% solvent B over 68 min was employed followed by 25–35% B over 5 min, an increase to 80% B over 2 min, a 2 min hold at 90% B, return to 3% B over 1 min, and 8 min of re-equilibration. The eluent from the HPLC was directly coupled to the DuoSpray source of the TripleTOF 6600 MS. The ionspray voltage was set to 5,500 V; the curtain gas was set to 138 kPa (20 psi), and the ion source gas 1 and 2 (GS1 and GS2) were set to 103 and 138 kPa (15 and 20 psi). The heated interface was set to 150°C. The discovery data files of individual technical replicates of either trypsin or chymotrypsin digested GW and MW samples were searched using ProteinPilot v5.0.3 with Paragon Algorithm (SCIEX) against a FASTA file consisting of Triticeae tribe proteins from UniProt-KB [accessed 02/2021 supplemented with additional translated gene models from the IWGSC RefSeq v1 Assembly (2), as well as those listed on the common Repository of Adventitious Proteins (thegpm.org/crap)]. The FASTA file contained 817,698 protein sequences.

### Targeted Proteomics

Reduced and alkylated tryptic and chymotryptic peptides were chromatographically separated on an Exion LC-40AD UHPLC system (SCIEX) and analyzed on a 6,500+ QTRAP mass spectrometer (SCIEX). Data acquisition was achieved using scheduled multiple reaction monitoring (sMRM) scanning experiments using a 60 s detection window for each MRM transition and a 0.3 s cycle time.

To build the MRM method, a FASTA file containing all identified proteins was imported into Skyline (42), all fully tryptic peptides sized between 6 and 30 amino acids were selected, and repeated peptides removed. All fully chymotryptic peptides between 6 and 30 amino acids were selected in independent experiments. Initially, five transitions were selected per peptide in an unscheduled MRM assay and assessed on both GW and MW samples. Those peptides where at least three transitions reproducibly co-eluted at the expected retention time (RT) without interference were then selected for inclusion in scheduled MRM assays. These were divided across several separate transition lists, such that all data was recorded with a 60 s detection window and maximum cycle time of 0.3 s. Precursor ions where three or more transitions had consistent RT, intensity over 1,000 cps, and a signal to noise ratio (S/N) >5 were kept and the three most intense transitions were selected for subsequent quantitative experiments. In this way, a total of 768 tryptic and 175 chymotryptic peptides that were unique to one protein were monitored, as well as 263 tryptic and 109 chymotryptic peptides that were present in more than one protein. Data was collected on four technical replicates of GW and MW. Peptide peak area data was exported from Skyline and analyzed (Graphpad Prism v8).

To quantify the relative abundance of individual gluten protein groups, peak areas of both unique and non-unique peptides were summed. Proteins were mapped to the wheat genome using the tBLASTn function of CLC Main Workbench v20.0.4 (Qiagen, Denmark), and multiple proteins mapping to the same gene were interpreted as different alleles of the same gene. Quantified MRM peptides were then allocated to protein groups according to the proteins in which they were found. Peptides occurring in proteins from multiple groups were labeled Multiple/Mixed. The significance and fold change of these protein groups were graphed using VolcaNoseR software (43).

A high sequence similarity between gluten proteins meant many peptides were observed that were common to multiple gluten proteins, making it impossible quantify all proteins using unique peptides. To overcome this, peptides quantified via MRM were allocated to gluten protein groups that were quantified using unique peptides for GW and MW, revealing the relative abundance of protein groups in these samples. To do this, all peptides quantified were mapped to the wheat genome (2), and using a combination of sequence alignments, the presence of Pfam domains (PF13016, PF03157, PF00234), and manual checking of the matching proteins were allocated to one of the following protein groups: α-gliadins, ALPs, ATIs, γ-gliadin, HMW-GSs, LMW-GSs, and ω-gliadins. Where the proteins had two or more peptides from multiple protein groups they were defined as “mixed.” While the ATIs and ALPs are not canonical gluten proteins, several of the ALPs can function as nutrient reservoir proteins, and the ATIs exhibit some allergenicity making them relevant to this investigation.

To quantify protein groups, the monitored peptides were mapped to the identified gluten protein sequences and non-gluten protein families with immune-reactive properties using 100% sequence matching in the Motif search algorithm in CLC Genomic Workbench v21.0.3 (Qiagen, Denmark), and group specific peptides were identified. Quantitative data on all chymotryptic and tryptic peptides were combined, and the abundance of each peptide in each replicate was normalized to the average seen across all replicates from both GW and MW. Graphs were generated in Graphpad Prism v8.

### Gene Enrichment Analysis

GO enrichment analysis was performed to test for the downregulation of certain classes of proteins in GW. Those proteins present in both GW and MW were excluded so that only proteins unique to GW or MW were analyzed for enriched GO terms. GW- or MW-specific proteins were then mapped to the wheat genome using CLC Genomic Workbench v21.0.3 (Qiagen, Denmark), and lists of their corresponding wheat gene identifiers were pasted into g:Profiler (biit.cs.ut.ee/gprofiler/gost) for GO overrepresentation analysis.

### Epitope Mapping

The peptides identified at 1% FDR in discovery proteomics were searched for known CD related T cell epitopes [Ludvig M (44)], baker's asthma, and wheat allergy related epitopes collected from the Immune Epitope Database and Analysis Resource (www.iedb.org) using the Motif search algorithm in CLC Genomic Workbench v21.0.3 (Qiagen, Aarhus, Denmark). Additionally, peptides recognized by commercial ELISA kits using R5 and G12 monoclonal antibodies were also mapped to the protein and peptide sequences. Hits with 100% sequence identity were kept in the analysis. The peptides monitored in MRM assays were also mapped to the same protein list, and the overlap between CD epitopes and the monitored peptides were determined. Monitored peptides that contained an entire epitope in their sequence were selected and quantified in GW and MW to give a relative measure of potential immune reactivity.

### Protein and ELISA Measurement

Protein estimations were performed using a Coomassie dye binding protein assay using Bradford reagent (Sigma-Aldrich, St Louis, USA) following the manufacturer's instructions. Measurements were made at 595 nm using a Varioskan LUX microplate reader (Thermo Scientific, Scoresby, Australia). Bovine serum albumin (BSA) standard was used in the linear range 0.125–1.5 mg/mL.

Diluted wheat extracts were analyzed by sandwich ELISA using the Ridascreen Gliadin (R-Biopharm, Darmstadt, Germany). The analytical protocols provided by the kit manufacturer were strictly followed. Each of the samples was extracted using the extraction Cocktail (R7006/R7016, R-Biopharm) recommended by the manufacturer for optimal gluten extraction and measured on the using Varioskan LUX microplate reader (Thermo Scientific) in duplicate on a single ELISA plate alongside the supplied standards (representing a gluten concentration of 5–80 mg/kg). The results of absorbance readings were analyzed according to the kit manufacturer's instructions using cubic polynomial regression for the standard curve. The data analyses were performed using Microsoft Excel.

## Results

### Discovery Proteomics

To identify the proteins in the GW and MW samples, combined database searches were performed on the discovery proteomics datasets. The numbers of distinct proteins identified at 1% global false discovery rate (FDR) excluding identifications against the common contaminants (cRAP database) are summarized in Table 1. This information is generated from the reports available at https://doi.org/10.25919/fr8e-k267, processed with the Protein Alignment Template v3.002 beta (SCIEX) and manual curation.

**Table 1:** Numbers of distinct proteins and of gluten and ATI proteins identified at 99% confidence in trypsin and chymotrypsin data.

	**Trypsin**	**Chymotrypsin**	**Combined**
**GW total**	**285**	**139**	**360**
GW gluten	76	75	126
α-gliadin	6	11	15
ATI	24	16	29
Avenin-like protein	11	5	12
γ-gliadin	11	8	18
HMW-GS	13	9	17
LMW-GS	11	23	32
ω-gliadin	0	3	3
GW non-gluten	209	64	234
**MW total**	**360**	**151**	**448**
MW gluten	73	93	138
α-gliadin	6	16	19
ATI	27	19	36
Avenin-like protein	9	6	10
γ-gliadin	10	16	23
HMW-GS	9	9	13
LMW-GS	11	23	32
ω-gliadin	1	4	5
MW non-gluten	287	58	310
**GW and MW combined**	**440**	**179**	**541**

Of the 541 proteins identified in both GW and MW (Table 1), more were identified in tryptic digests (440) than chymotryptic digests (179). Greater representation of α- and γ-gliadins and LMW-GS was achieved using chymotryptic digests, while more ATIs, ALP-derived, and non-gluten proteins were identified in tryptic digests (Table 1).

**Figure 1:**
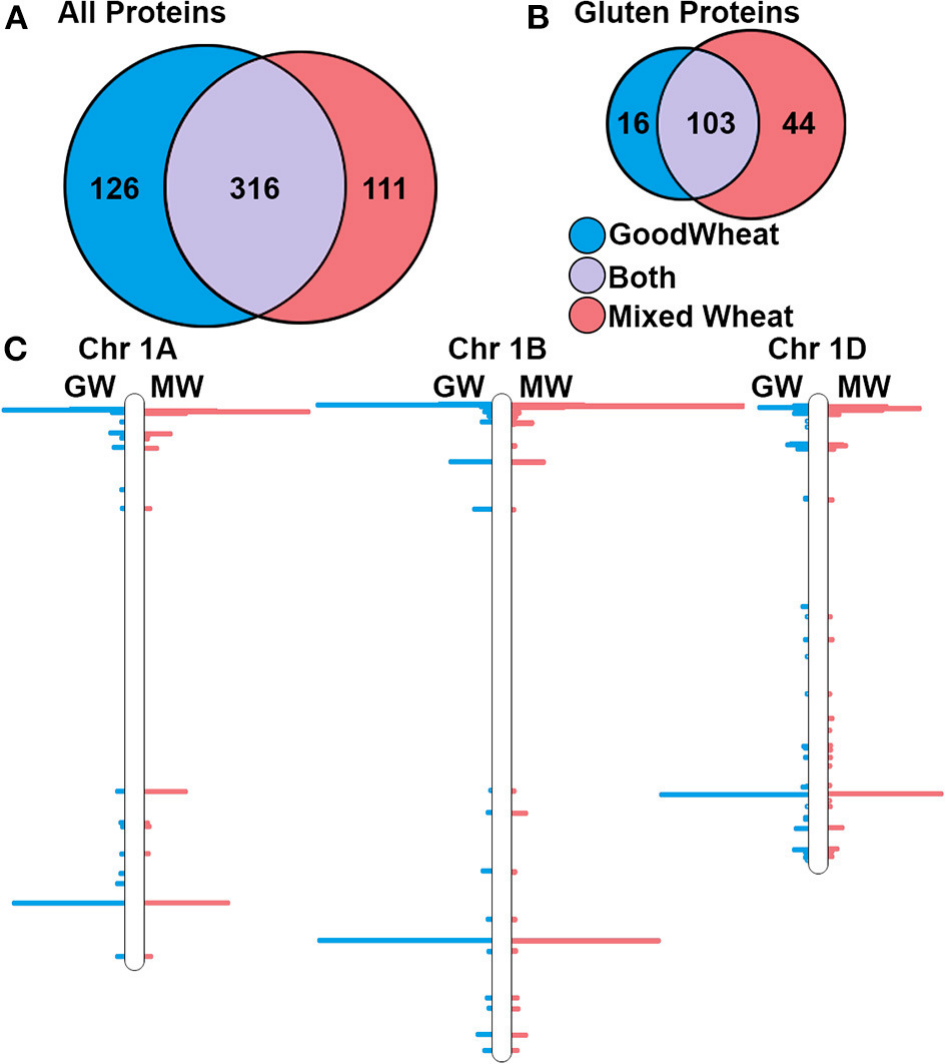
Summary of total and gluten-like proteins identifications at 1% FDR and their corresponding chromosomal locations. **(A)** Venn diagram showing the total number of proteins detected within the GW and MW datasets. **(B)** Detected gluten proteins in MW and GW. **(C)** Locations of genes for detected proteins on wheat chromosomes 1A, 1B, and 1D.

Considering trypsin and chymotrypsin data together enables a more complete comparison of the GW and MW proteomes. Together, there were 360 distinct proteins identified in GW and 448 in MW, with an overlap of 267. The higher number in MW reflects the genetic diversity of the multiple wheat varieties that are present. In GW, 126 of identified proteins were gluten-like proteins (35%) and in MW this number was 138 (31%). Notably, the GW and MW proteomes share 99 (60%) of the 165 detected gluten proteins. The numbers of proteins identified are compared in Figures 1A,B. To identify the chromosomal position of these proteins within the wheat genome protein, sequences were mapped to the IWGSC wheat genome assembly version 1 (2), and the number of peptides observed per 1 million base pairs (Mb) bins was determined. This revealed clusters of detected proteins in all known storage protein gene loci regions of the genome, corresponding to γ- and ω-gliadins, LMW-GS, and HMW-GS (3) on chromosome group 1 and α-gliadins on chromosome group 6. Figure 1C shows the location of the peptides detected superimposed on chromosomes 1A, B, and D, respectively. Though non-gluten proteins were also detected across all wheat chromosomes, there were no large-scale chromosome region changes observed in GW and MW, indicating the potential for gene expression of gluten proteins in GW.

### Targeted Proteomics

**Figure 2:**
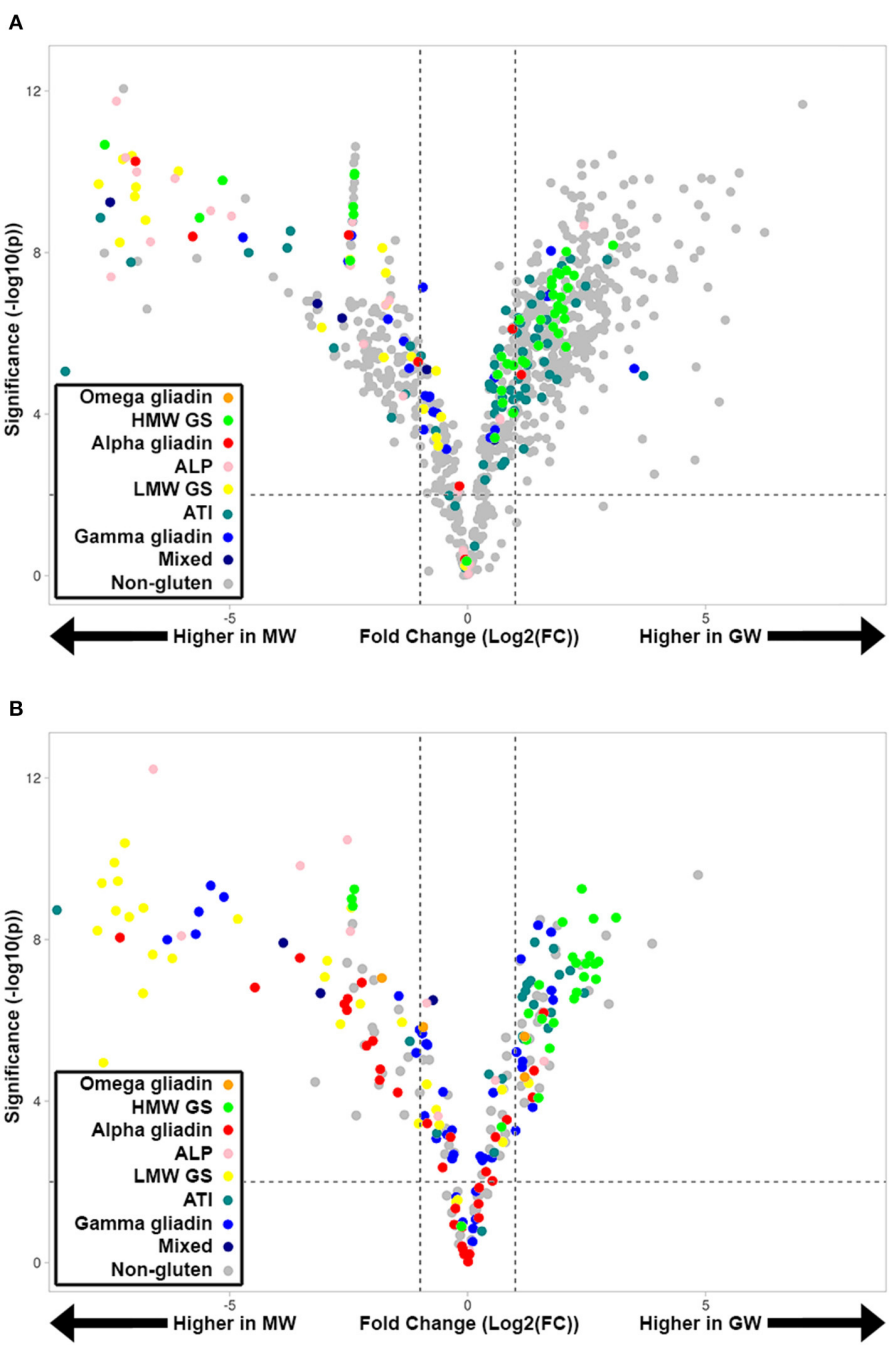
Volcano plots showing quantified tryptic **(A)** and chymotryptic **(B)** peptides in GW and MW samples colored according to gluten group. A fold-change of 2 is indicated by the dashed vertical lines [Log_2_(FC) = ±1]. Peptides above the horizontal dashed line have a significant change in abundance between GW and MW (*p*-value <0.01 [–log_10_(*p*) >2].

To investigate the quantitative changes across GW and MW wheat samples, LC-MRM-MS-based quantitative assays were developed for all peptides confidently identified in the discovery proteomics experiment (Figure 2). A total of 189 tryptic peptides and 170 chymotryptic were targeted. While 84 tryptic and 55 chymotryptic peptides were uniquely present, i.e., in only one protein isoform, many of the peptides monitored by MRM occur in multiple protein isoforms and therefore reflect the relative abundance of more than one protein. While LC-MRM-MS reveals peptide relative abundance, using this information to quantify proteins by combining the constituent peptides is confounded by both the presence of repeated peptides and differential ionization efficiency of various peptides. We therefore categorized peptides into groups that reflect the abundance of major allergen types and did not quantify specific proteins. This revealed the fold-change and significance of tryptic (Figure 2A) and chymotryptic peptides (Figure 2B) peptides between GW and MW. HMW-GS and ATI peptides tend to be higher in GW than MW, and many tryptic “non-gluten” peptides are higher in GW. Similarly, many LMW-GS, ALP, and α-gliadin peptides are lower in GW than MW.

**Figure 3:**
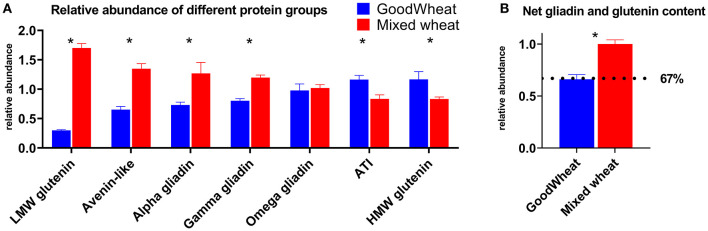
Relative abundance of different gluten or ATI protein groups **(A)**. Quantitation was performed based on all detectable peptides from proteins classified to these groups. Error bars indicate SEM, and significant differences are indicated by asterisk. Adding together the LMW and HMW glutenins, and α-, γ-, ω-gliadins gives the net gliadin and glutenin content **(B)** which equated to GW having an estimated 67% of the gluten content of MW (dotted line).

The normalized peak area for all peptides belonging to each protein group were then summed to compare the overall abundance of each protein group (Figure 3). Importantly, GW showed significantly lower abundance of LMW-glutenins, α-gliadins, and γ-gliadins, but showed an increase in HMW-glutenins relative to MW. GW also showed significant decreases in ALPs and increases in ATIs. Changes in net ω-gliadin abundance were not significant. The net change in canonical gluten content can be obtained by adding together the gliadins and glutenins (LMW-GS, HMW-GS, α-, γ-, ω-gliadins), showing that GW has 67% the relative gluten protein abundance as MW (Figure 3B).

### Gene Set Enrichment Analysis

**Figure 4:**
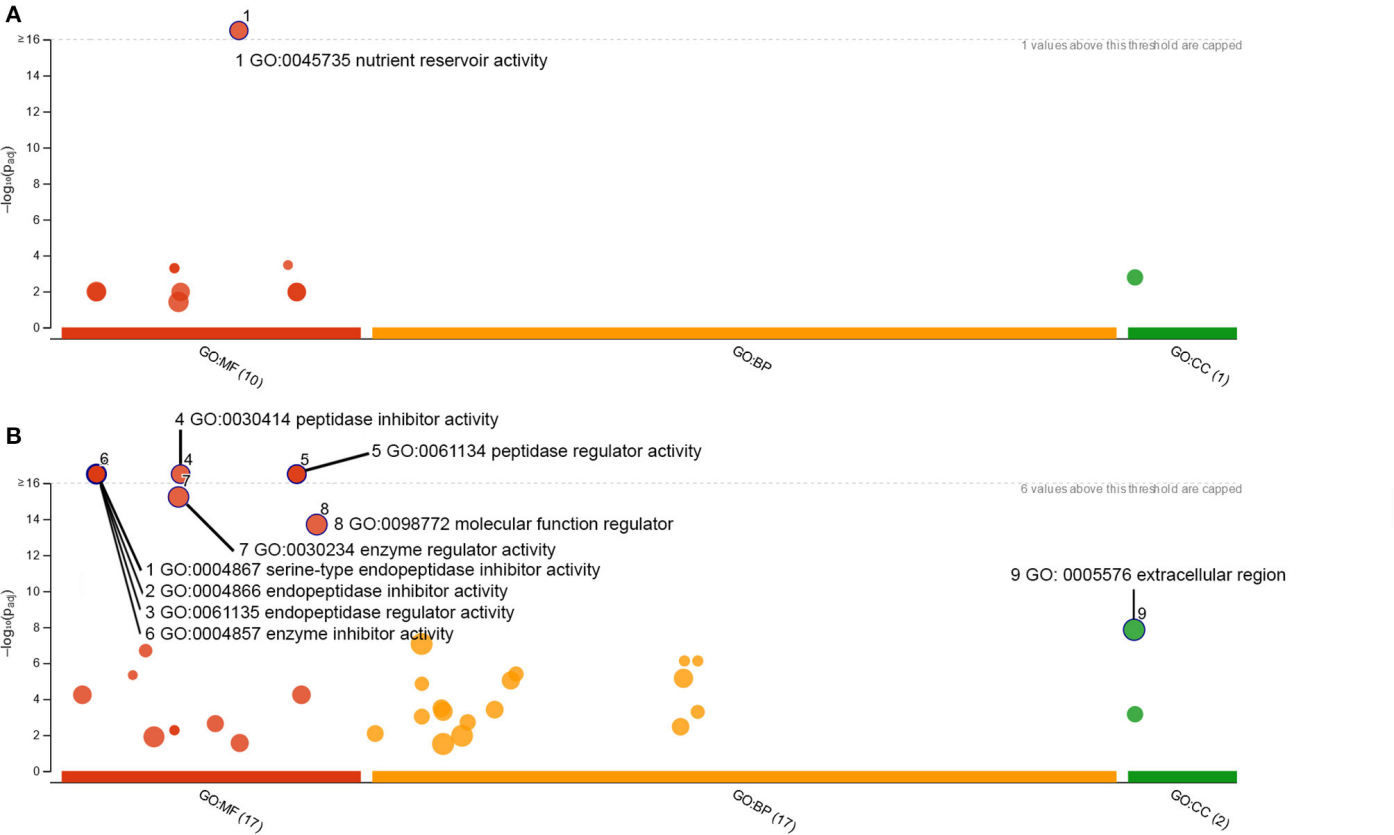
GO enrichment analysis of proteins showing ≥2-fold increase in: MW **(A)**; or GW **(B)**.

To understand the enrichment of protein classes within the individual wheat samples, GO enrichment analysis was performed using g:Profiler on those proteins detected with a fold change ≥2 in MW and GW as shown in Figure 2. Proteins in MW showed predominant enrichment for nutrient reservoir activity (GO:0045735, Figure 4A). GW proteins showed enrichment of several classes of enzyme inhibitor and regulators, as well as enrichment of proteins localizing to the Extracellular Region (GO:0005576) cellular compartment indicating the compensation mechanism for the expression of non-gluten proteins. There was no enrichment of nutrient reservoir activity (Figure 4B).

### Epitope Mapping

**Figure 5:**
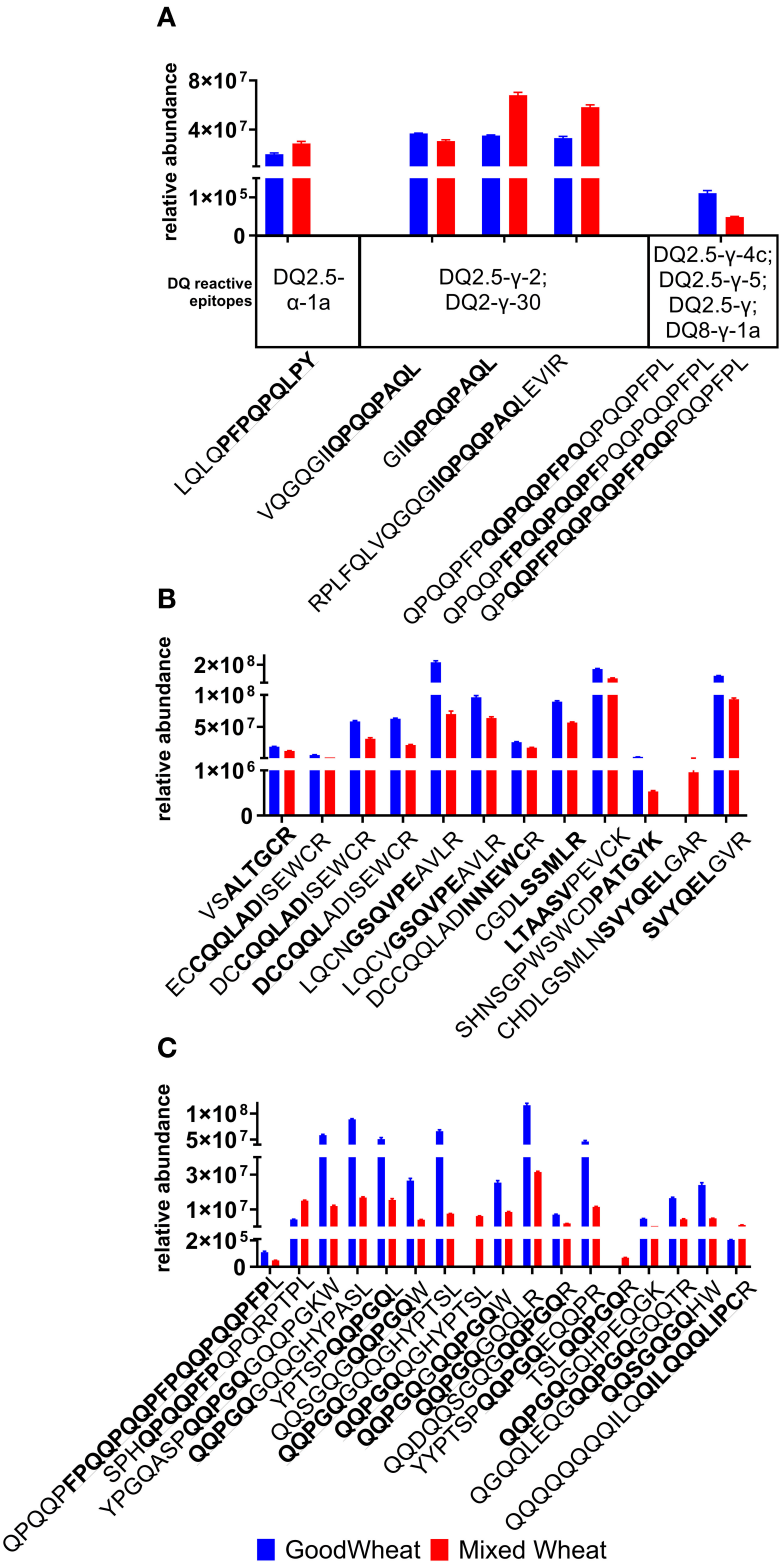
Relative abundance of immune reactive epitopes in peptides quantified in MRM assays, reported as mean and standard error. Epitope sequences are highlighted in bold within the peptide sequence: **(A)** Coeliac disease HLA-DQ reactive epitopes; **(B)** Bakers' asthma; **(C)** Wheat Allergy plus peptide QQQQQQQQILQQILQQQLIPCR which contains the epitope QILQQQLIPC antigenic for wheat-dependent exercise induced anaphylaxis. When calculating the total, duplicate peptides where multiple epitopes were detected were only counted once.

To explore the potential immune reactive nature of proteins detected in GW compared to those in MW, peptides identified in the discovery data that contained full-length immune reactive epitopes were quantified (Figure 5). Known immunogenic regions within quantitated MRM peptides are quantified, including HLA-DQ T cell epitopes for CD patients (Figure 5A), baker's asthma epitopes (Figure 5B), and wheat allergy-related epitopes (Figure 5C). It should be noted that these represent a small subset of the known immune reactive epitopes. The discovery analysis results (Supplementary Table 1) indicate the presence of additional epitopes that were not quantified with MRM. There were six complete HLA-DQ T cell epitope sequences observed in a total of 25 peptides, nine BA epitopes in 12 peptides, two WA epitopes in 16 peptides, and one WDEIA epitope in one peptide. Overall, HLA-DQ reactive epitopes in GW were present at 67% the relative abundance of MW. BA-reactive epitopes were also more abundant in GW at 180% that of MW.WA-reactive epitopes were also more abundant in GW at 379% the level seen in MW. Only one wheat dependent exercise-induced anaphylaxis epitope was observed, which was notably lower in GW at 17.7% the level seen in MW.

The protein content of MW and GW were evaluated and did not show a significant difference at 0.84 and 0.88 mg/mL, respectively. The gluten content was also evaluated by R5 ELISA and it was interesting to note that GW revealed a 39% higher gluten content than MW, an unexpected result given the overall decrease in gluten peptides detected by LC-MS.

## Discussion

The current study used complementary high sensitivity LC-MS techniques to identify gluten proteins and to monitor the relative abundance of gluten and allergenic wheat proteins in a recently developed wheat product (GoodWheat, GW) in comparison to a wheat sample mixed from equal amounts of nine commercial cultivars (Mixed wheat, MW). Peptides from gliadin and glutenin proteins were present in GW at 67% of the abundance of the MW control, indicating an average decrease of 33% (Figure 3B). This is complemented by our analysis of intact HLA-DQ reactive epitopes in the monitored peptides which were 67.3% as abundant in GW as MW (Figure 5A). While this may reduce but not remove the antigen content of GW, it is accompanied by an increase in peptides known to be related to Baker's asthma and wheat allergy, at 180 and 379%, respectively. The overlap between immunogenic DQ epitopes with peptides detected in discovery data, and quantified in MRM is presented in Supplementary Table 1. While the use of LC-MRM-MS in this work enabled the quantitation of gluten proteins and highlights its utility in grain protein research with specific reference to gluten, future studies should focus on using complementary extraction buffers to understand more about changes in the GW grain proteome.

In contrast to the MRM analysis that revealed an overall lower gluten content in GW than MW (Figure 3), the R5 ELISA estimate of gluten content revealed a gluten content ~39% higher in GW than MW. The slightly elevated protein content (5%) in GW compared to MW would account for a minority of the observed difference. The elevated ELISA measurement likely reflects an overall increase in the ratio of R5 epitope per unit of protein. The choice of reference material, in particular the ratio of gliadin to glutenin, is known to affect measurements of gluten by ELISA even in simple food matrices (45), and kits that use different primary antibodies will yield different measurements of gluten (46) because of the specificities and sensitivities of the primary antibody (47). Future analyses should investigate the gluten content of GW using alternative ELISA kits or gluten protein quantitation employing fractionation (RP-HPLC or size-exclusion chromatography) protocols.

Important trends were seen in specific gluten protein types (Figures 2, 3), as the LMW-GSs, α-, and γ-gliadins are lower in GW, while the HMW-GSs were significantly more abundant. HMW glutenins contribute more to bread's elastic properties than other gluten proteins due to their relative size and ability to form large polymers (48). Their higher relative abundance in GW indicates that they in part compensate for the lower abundance of gliadins and LMW glutenins (49). Additionally, the level of immune response elucidated by HMW glutenins in CD is significantly lower compared to the α-, γ-, and ω-gliadins and LMW glutenins (40), making their increase less relevant to CD, however it has important implications for WA and BA. Along with ATIs, which were also significantly higher in GW, the higher HMW glutenin content in GW brings more allergenic epitopes related to WA and BA. This is reflected in Figure 5 as the allergenic epitopes recognized by different B and T cell types are increased by an overall 53.5%.

Interestingly, the ALPs were also present in significantly lower amounts in GW than MW. While named for their resemblance of oat avenins (50), these seed storage proteins share sequence similarity and secondary function with γ-gliadins and LMW glutenins (51). They contribute both to allergenicity (52) and bread dough quality (53), and contain one or two gliadin (PF13016) domains. ALPs also contain CD-related B cell epitopes (3), and their downregulation is important for CD toxicity.

The symmetry of the volcano plot (Figure 2) indicates the net decrease in gluten protein and ALP content is accompanied by compensatory expression of other proteins within the grain. GO enrichment analysis of the GW proteome revealed enzyme inhibitors and regulators that are enriched in GW which was also confirmed by the gene set enrichment analysis showing cysteine-rich proteins are overrepresented in the upregulated proteins in GW. Most of these proteins have a defense related function and were upregulated in lieu of proteins with a canonical “nutrient reservoir activity” GO MF annotation (Figure 4).

Our proteogenomic analysis indicates that there is no evidence of large-scale chromosome deletions or absence of storage protein gene clusters (Figures 1B,C) on chromosome group 1 and 6 in GW. While antibody-based assays or classical Osborne fractionation were not performed and thus represents a limitation of the present work, gluten proteins were present in both GW and MW and simply expressed at different levels (Figure 3). This would suggest the novel GW variety expresses less gluten proteins due to gene regulation at a transcriptional or post-transcriptional level. There are several known mechanisms implicated in seed development and gluten protein expression that may be at play. One is the *LYS3* gene that encodes the transcription factor Prolamin Binding Factor (PBF). PBF is expressed early in seed development, and suppresses seed growth by reducing the expression of developmental and starch metabolism genes (54). Wheat *lys3* mutants have been reported to contain lower levels of gliadins and LMW-GSs (21), which matches our results as shown in Figure 4. A barley variety with *lys3a* mutation causing it to not express C-hordein (a class of barley gluten) was used in a breeding program to derive an “ultra-low gluten” barley variety (30, 55), showing it is compatible with selective breeding. These low-gluten PBF mutant lines exhibit increased expression of lysine-rich genes that are otherwise related to developmental processes during germination (30). While it is possible that GW uses *lys3* mechanisms to regulate gluten protein expression, using solely the proteomic information presented in this study we cannot conclusively determine the targeting of *lys3* regulation.

In conclusion, the use of discovery and targeted proteomics-based experiments has enabled the detection and quantitation of gluten and additional allergenic proteins present in the GW and MW samples. This study revealed a 33% decrease in gluten-like proteins in GW and the compensatory expression of non-gluten proteins within MW samples that tend to have enzyme inhibitor or regulator activity GO terms. This study affirms that, as stated by the manufacturer, GW is not compatible with a gluten-free diet. Epitope mapping revealed a reduction in gluten protein-specific epitopes; however, there was an increase in epitopes related to baker's asthma and wheat allergy in GW wheat in comparison to MW. Additionally, the chromosomal level analysis of detected proteins showed no significant differences between GW and MW. Future studies focusing on integrating LC-MS/MS results with clinical measurements would be needed to investigate the nutritional benefits of GW. Overall, the current study exemplifies the use of proteogenomic approaches as a tool to explore the safety and/or health benefits of wheat varieties targeted toward consumers with wheat-related disorders.

## Data Availability Statement

The datasets presented in this study can be found in online repositories. The names of the repository/repositories and accession number(s) can be found below: https://doi.org/10.25919/fr8e-k267.

## Author Contributions

MN-W: sample preparation, LC-MS data collection, data analysis, and manuscript preparation. AJ and UB: data analysis and manuscript preparation. MC: project concept and design and manuscript preparation. All authors contributed to the article and approved the submitted version.

## Conflict of Interest

The authors declare that the research was conducted in the absence of any commercial or financial relationships that could be construed as a potential conflict of interest. The reviewer KS declared a past co-authorship with one of the authors, MC, to the handling editor.

## Publisher's Note

All claims expressed in this article are solely those of the authors and do not necessarily represent those of their affiliated organizations, or those of the publisher, the editors and the reviewers. Any product that may be evaluated in this article, or claim that may be made by its manufacturer, is not guaranteed or endorsed by the publisher.
